# Effect of Microwave Irradiation on Lead Adsorption Properties of Vermiculite with Different Particle Sizes

**DOI:** 10.3390/ma17164152

**Published:** 2024-08-22

**Authors:** Yunzhu Chen, Hongjuan Sun, Tongjiang Peng, Wenjin Ding, Hongmei Yin

**Affiliations:** 1 Key Laboratory of Ministry of Education for Solid Waste Treatment and Resource Recycle, Southwest University of Science and Technology, Mianyang 621010, China; chenyunzhu@xcc.edu.cn (Y.C.); tjpeng@swust.edu.cn (T.P.); dingwenjin@swust.edu.cn (W.D.); 15281194971@163.com (H.Y.); 2 Department of Resources & Environment, Xichang University, Xichang 615000, China; 3 Institute of Mineral Materials and Application, Southwest University of Science and Technology, Mianyang 621010, China

**Keywords:** vermiculite, microwave expansion, particle size, lead adsorption

## Abstract

The expansion of vermiculite using microwave irradiation is an environmentally friendly and efficient method that can enhance the material’s adsorption performance. This study investigated the microwave irradiation of vermiculite with five different particle sizes (4/2/1/0.5/0.2 mm) and found that the adsorption capacity for Pb^2+^ increased with larger particle sizes. The equilibrium adsorption capacity reached 15.98 mg/g at 4 mm, representing a 45.01% improvement compared to 0.2 mm. The pseudo-second-order kinetic model effectively described the adsorption kinetics. No significant differences were observed in the specific surface area and pore size distribution of all samples. Thermogravimetric quantitative analysis revealed that larger particle sizes retained interlayer water more effectively. As the particle size decreased, the interlayer water content generally showed a decreasing trend. Fourier-transform infrared spectroscopy analysis also indicated that the -OH groups in larger particle sizes exhibited higher stability. The results suggest that the high content and stability of -OH groups may be key factors in the enhanced adsorption performance for Pb^2+^. This provides new insights for the preparation of environmentally friendly adsorbent materials rich in hydroxyl groups.

## 1. Introduction

Vermiculite is an excellent material for soil improvement and pollutant adsorption [1,2]. The addition of vermiculite to soil contaminated with heavy metals such as copper, chromium, and nickel can significantly reduce the accumulation of these metals in plants [3]. A novel adsorbent, prepared by combining vermiculite with yeast, effectively reduces the toxicity of Cd^2+^ [4]. Expanded vermiculite can efficiently recover the Dy, thereby minimizing resource waste [5]. The use of hydrogen peroxide to expand vermiculite can effectively increase the concentration of exchangeable cations in clay minerals, thus enhancing the adsorption capacity for Pb^2+^. These advantages stem from the larger specific surface area, higher concentration of exchangeable cations, and elevated surface potential of the expanded vermiculite. As a result, an increasing number of researchers are utilizing expanded vermiculite to develop high-performance adsorbent materials.

The three common expansion methods include high-temperature, chemical, and microwave methods. High temperatures can damage the structural integrity of vermiculite, reducing its durability and limiting its applications in environmental protection [6,7]. Chemical expansion can effectively preserve the structure of vermiculite, particularly the -OH functional groups on its surface [8]. However, the long reaction time poses challenges for practical production [9]. Microwaves propagate at the speed of light and transfer energy directly to the material in a low-energy and efficient manner (Microwave irradiation causes the rotation of water molecules and the reversible diffusion of ions, which in turn leads to rapid heating and evaporation of water without significantly increasing the temperature of the solid materia) [10,11]. Sun et al. [12] found that at a microwave power of 480 W, it took only 1 min to achieve a colloidization rate of 98% in organic montmorillonite, with the interlayer spacing increasing to 2.65 nm, significantly reducing energy consumption. The vermiculite subjected to microwave irradiation exhibited excellent expandability, a low water loss rate (less than 2%), and negligible external surface water absorption [13], making it more effective in adsorbing environmental pollutants. Therefore, expanded vermiculite prepared through microwave irradiation shows promising potential for environmental remediation.

There have been intriguing findings related to the use of microwave-expanded vermiculite. For instance, the moisture content of interlayer water in vermiculite affects the efficiency of microwave exfoliation. Liu et al. [14] found that Mg^2+^-modified vermiculite absorbs microwave energy more effectively, primarily due to the hydrating reaction of Mg^2+^, which increases interlayer moisture content. The type and composition of interlayer cations determine the equilibrium moisture content and interlayer spacing of clay minerals [15]. Ruiz et al. [16] discovered that the water mobility in larger vermiculite particles is lower than that in smaller particles. However, there is limited reporting on the changes in adsorption properties of vermiculite of different sizes following microwave treatment. Lead, as one of the most widely distributed toxic metals globally, has received considerable attention [17]. The study of lead adsorption using expanded vermiculite holds significant scientific and practical application value. Based on this, this paper will select vermiculite of varying sizes for microwave irradiation treatment, followed by adsorption experiments of Pb^2+^ from aqueous solutions, in order to investigate the changes in adsorption performance and the mechanisms involved.

## 2. Material and Methods

### 2.1. Materials and Sample Preparation

For this study, vermiculite sourced from Xinjiang Yuli was chosen, characterized by diameters ranging from 2 to 4 mm and thicknesses between 0.3 and 0.5 mm. This particular vermiculite exhibits a regular interlayer structure with a 1:1 ratio. The chemical composition of the vermiculite was presented in Table 1. Additionally, the bulk density of the vermiculite was measured to be 898.46 kg/m^3^.

**Figure 1:**
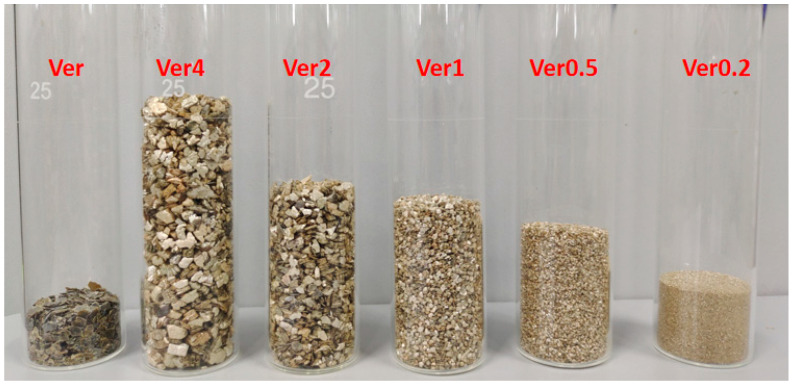
Comparison of expansion effect of vermiculite with different particle sizes after microwave irradiation.

Initially, five types of vermiculite with the same weight and different particle sizes (4, 2, 1, 0.5, 0.2 mm) were subjected to microwave irradiation at a power of 800 W for a duration of 10 min as shown in Figure 1. Subsequently, the expanded vermiculite was finely ground to a particle size of 0.15 mm. Each of these processed samples was assigned a distinct label, namely Ver4, Ver2, Ver1, Ver0.5, and Ver0.2, respectively. The unprocessed mineral was designated as Ver. All of the aforementioned samples were stored in bags.

The expansion rate K is measured by the change in the bulk density. As shown in Equation (1). The bulk density was measured by the method in the national standard JC/T 441-2009 [18].

(1) $$K=\frac{\text{Experimental}\;\text{group}\;\text{bulkdensity}}{\text{Ver}\;\text{bulk}\;\text{density}}$$

**Figure 2:**
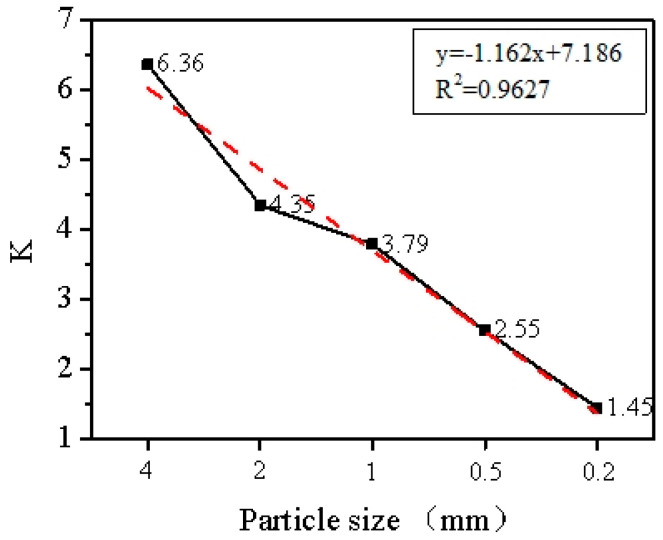
Expansion effect of microwave irradiation on vermiculite of different particle sizes.

Figure 2 shows the relationship between expansion rate (K) and particle size after microwave irradiation. The data shows a negative correlation between these two variables. As the particle size decreases, the value of K decreases (R^2^ = 0.9627). Specifically, K was 6.36 and 1.45 for particle sizes of 4 mm and 0.2 mm, respectively. The smaller the particle size, the poorer the expansion effect.

### 2.2. Adsorption Experiment

The procedure was as follows: 5 mg of the pre-treated sample was placed into a 100 mL centrifuge tube. Then, 50 mL of the Pb^2+^ solution was added, and the tube was shaken thoroughly. After centrifuging at high speed (8000 rpm), solid-liquid separation was performed using membranes with a pore size of 0.45 µm, and the Pb^2+^ concentration in the filtrate was determined. Three parallel samples were made under the same conditions, and the results were averaged.

The concentration of Pb^2+^ solution was determined by ICP-OES. The equilibrium adsorption amount (*q_e_*) of the sample was calculated according to Equation (2), where *q_e_*, *C*_0_, *C_e_*, *V*, *m* represent the equilibrium adsorption amount, the initial concentration, the equilibrium adsorption concentration, the volume of Cd solution, and the sample mass.

Kinetic studies are indispensable for describing the adsorption process, and adsorption kinetics are necessary to further explain the heavy metal removal mechanism. Here pseudo-first-order (Equation (3)) and pseudo-second-order (Equation (4)) kinetic models were used to fit the experimental data for Pb^2+^. Where *q_t_* represents the removal amount towards Pb^2+^ at time *t*, *q_e_* (mg/g) is the removal amount towards Pb^2+^ at equilibrium; *k*_1_ and *k*_2_ represent the rate constants.

(2) $$q_{e}=\frac{\left(C_{0}-C_{e}\right)v}{m}$$

(3) $$\ln\left(q_{e}-q_{t}\right)=lnq_{e}+k_{1}t$$

(4) $$\frac{t}{q_{t}}=\frac{1}{k_{2}q_{e}^{2}}+\frac{t}{q_{e}}$$

### 2.3. Measurement and Characterization Techniques

The micro-morphology and energy spectrum analysis of the samples were conducted using a scanning electron microscope (Ultra55, Zeiss, Jena, Germany). The surface functionality was analyzed using a Fourier-transform infrared spectrometer (Magna 550II, PerkinElmer Frontier, Waltham, MA, USA). Thermogravimetric analysis was performed with a thermogravimetric analyzer (SDT Q600, TA Instruments, New Castle, DE, USA). Additionally, the surface area and nitrogen (N_2_) adsorption-desorption characteristics of the samples were measured using the Brunauer–Emmett–Teller (BET) method with a Micromeritics ASAP 3020 (Norcross, GA, USA).

The concentration of exchangeable cations (CEC) in vermiculite was extracted with ammonium acetate solution (1 mole/liter, pH = 7), and then analyzed for K^+^, Na^+^, Ca^2+^, Mg^2+^, Sr^2+^, and Ba^2+^ using inductively coupled plasma optical emission spectrometry (ICP-OES, iCAP 5110 Full Spectrum Instrument, Shanghai, China) [19].

## 3. Results and Discussion

### 3.1. Adsorption Kinetics

**Figure 3:**
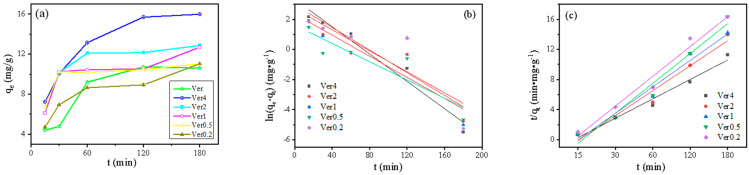
Isothermal adsorption data of Pb^2+^ by expanded vermiculite (**a**), curves fitted with pseudo—primary kinetic (**b**) and pseudo—secondary kinetic (**c**) models.

The adsorption rate of Pb^2+^ by different sizes of expanded vermiculite was investigated as a function of time and the results are shown in Figure 3. As can be seen from Figure 3a, the removal of Pb^2+^ from the samples was rapid during the first 60 min, followed by a slow-phase removal phase, and equilibrium was reached close to 3 h. The adsorption of Pb^2+^ by vermiculite after microwave irradiation was proportional to the size. The adsorption of Pb^2+^ increased in all samples except Ver0.2 which was lower than Ver.

The linear forms of Pb^2+^ were displayed in Figure 3b,c, and kinetics parameters were listed in Table 2, respectively. Based on the evaluation of linearity and R^2^ values of models, it showed that the pseudo first order reaction kinetic possessed poor correlations (R^2^ < 0.84), whereas the pseudo second order model was well-suited to describe the experimental data (R^2^ > 0.96). The better fitted results revealed that the adsorption process was controlled by the amount of the active sites on the adsorbent.

### 3.2. Characterization of Materials

**Figure 4:**
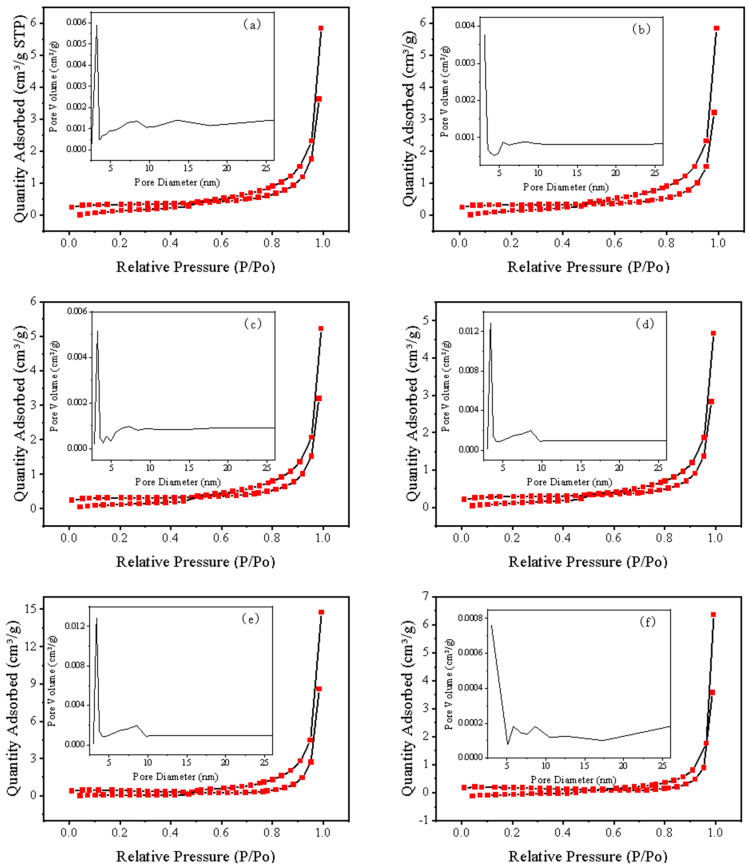
N_2_ adsorption-desorption isotherms and pore size distribution of expanded vermiculite with different sizes Ver (**a**), Ver4 (**b**), Ver2 (**c**), Ver1 (**d**), Ver0.5 (**e**), Ver0.2 (**f**).

First, the samples (powder) were subjected to N_2_ adsorption-desorption and pore size distribution analysis. A uniform pore size and a large specific surface area are important factors that enhance adsorption capacity [20]. The pore size distribution curve is shown in Figure 4. The pore sizes of all samples are primarily distributed around 3 nm, with no significant variation trend. Subsequently, the N_2_ adsorption-desorption curves were analyzed, revealing that all samples exhibit type IV adsorption isotherms, characteristic of mesoporous materials. At lower P/P_0_; values, the interaction between the adsorbent and the adsorbed gas is relatively weak, predominantly featuring monolayer adsorption. At higher relative pressures, an inflection point appears, indicating that aggregated molecules fill the pores. The hysteresis loop in the high-pressure region is nearly vertical, and the adsorption-desorption curves are almost parallel, suggesting a concentrated pore size distribution for the samples. There are no significant differences in the specific surface area and pore size among all samples. Therefore, the primary reason for the differences in adsorption capacity is not due to the increase in specific surface area or changes in pore size.

**Figure 5:**
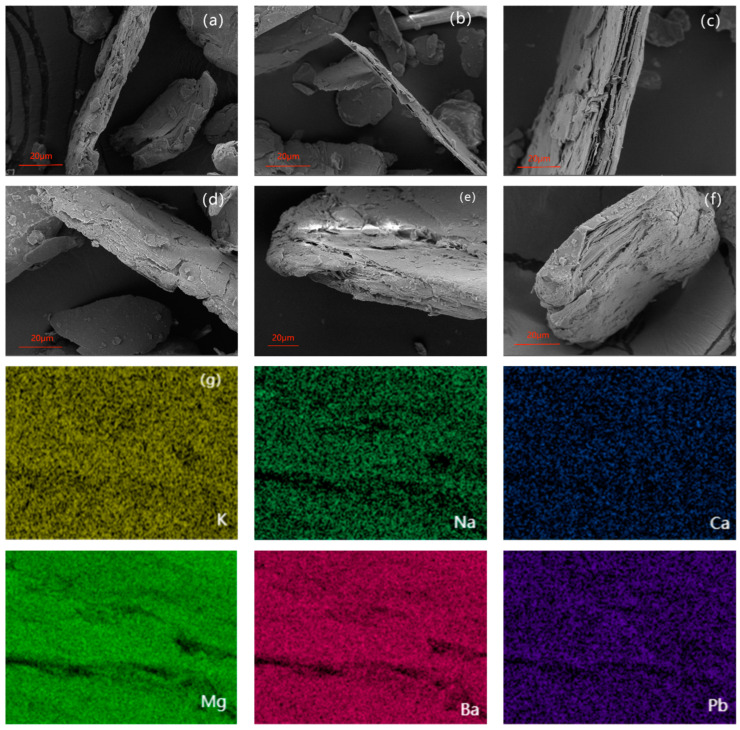
SEM of expanded vermiculite with different sizes, Ver (**a**), Ver4 (**b**), Ver2 (**c**), Ver1 (**d**), Ver0.5 (**e**), Ver0.2 (**f**), and elemental mappings (**g**) showing the distributions of K, Na, Ca, Mg, Ba, and Pb on Ver4.

The surface morphology and elemental mapping of the samples were studied by scanning electron microscopy as shown In Figure 5. The exfoliation degree of the samples showed a decreasing trend with decreasing particle size. Among them, Ver4 has the thinnest average lamellae. It confirms the trend of expansion rate. Although the degree of exfoliation of the samples varied, the changes in specific surface area and pore size distribution were not obvious. The kinetics of the adsorption process of expanded vermiculite is controlled by an external mass transfer model, in which ion exchange is also the main mechanism affecting the adsorption performance [21]. The exchangeable cations on the surface of vermiculite were dominated by K, Na, Ca, Mg, and Ba. Taking Ver4 as an example, it is shown in Figure 5g. The distribution of all elements after adsorption of Pb^2+^ is uniform rather than localized accumulation distribution. It indicates that there is an exchange interaction between Pb^2+^ and all five elements.

### 3.3. Adsorption Mechanisms Analysis

The cation exchange capacity (CEC) of the samples was studied, and the results are presented in Table 3. Compared to Ver, all samples exhibited a decreasing trend in CEC, which was inversely related to particle size, specifically Ver4 < Ver2 < Ver1 < Ver0.5 < Ver0.2. It is well known that vermiculite contains two types of water in the interlayer spaces [22]: interlayer water and structural water. Interlayer water can be further divided into bound water and free water [10]. Bound water can combine with cations to form hydrated cations, while free water, which is unaffected by cations, accounts for approximately half of the mass of interlayer water [23]. Changes in CEC inevitably affect the bound water content. Understanding the variation in bound water can help elucidate the mechanisms behind changes in adsorption capacity.

**Figure 6:**
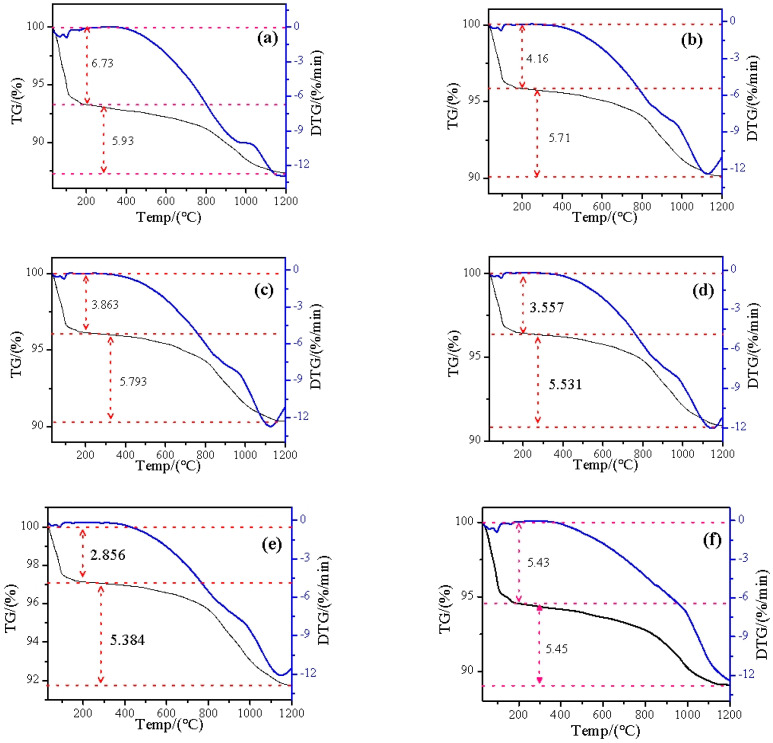
Thermogravimetric changes of expanded vermiculite with different sizes, Ver (**a**), Ver4 (**b**), Ver2 (**c**), Ver1 (**d**), Ver0.5 (**e**), Ver 0.2 (**f**).

A quantitative study of thermogravimetric analysis of the samples was conducted, with the results displayed in Figure 6. The loss of interlayer water and structural water is the primary cause of the mass changes in vermiculite. In the low-temperature range (30–200 °C), interlayer water was consumed, with free water evaporating more readily than bound water due to its unbound nature. In the high-temperature range (200–1200 °C), structural water was lost. Compared to the low-temperature phase, the changes in all samples during the high-temperature phase were relatively stable, with mass losses ranging from 5.38% to 5.93%. In contrast, the mass loss during the initial phase exhibited significant variation, ranging from 2.86% to 6.73%. After microwave treatment, the interlayer water content of vermiculite was ranked as Ver0.5 < Ver1 < Ver2 < Ver4 < Ver0.2. This result is inconsistent with the changes observed in CEC. With the exception of Ver0.2, the interlayer water content of the remaining samples increased with larger particle sizes, indicating that smaller particle sizes experienced greater mass loss than larger ones. Ghanem et al. [24] found that microwaves enhanced the stability of -OH groups, thereby improving the water retention capacity of citrus peels. Yan et al. [25] suggested that microwave radiation enhances the hydration capacity of materials. It can be inferred that larger particle sizes absorbed more microwave energy, increasing the stability of hydrated cations in the interlayer, making them less prone to evaporation at lower temperatures. The observed result is that the interlayer water content of larger particle sizes is higher than that of smaller ones.

**Figure 7:**
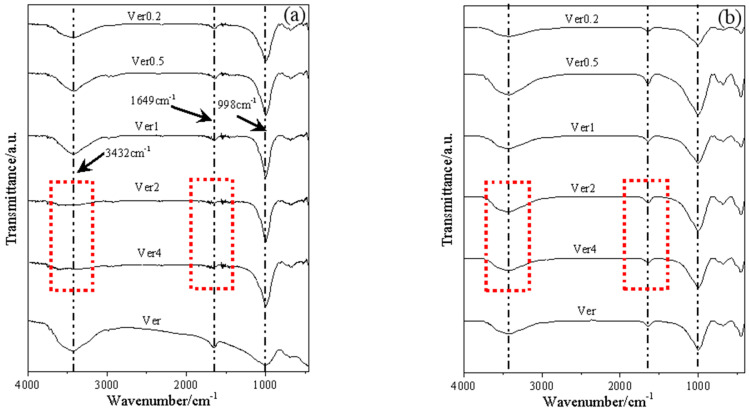
Fourier infrared spectra before adsorption of Pb^2+^ (**a**), Fourier infrared spectra after adsorption of Pb^2+^ (**b**).

The results of the FTIR analysis are presented in Figure 7. The peaks at 3432 cm^−1^ and 1649 cm^−1^ correspond to the vibrational absorption peaks of -OH groups on the surface of vermiculite [26]. Figure 7a shows the results for the samples after microwave treatment. It is evident that the peaks for Ver4 and Ver2 at 3432 cm^−1^ disappeared, and the intensity of the peak at 1649 cm^−1^ diminished. In comparison to Ver, no significant changes were observed in the other samples. Interestingly, the peaks that disappeared after Pb^2+^ adsorption reappeared, as shown in Figure 7b. The re-emergence of the hydroxyl vibrational peaks may be attributed to the samples being re-exposed to water during the adsorption process of Pb^2+^, leading to the acquisition of new interlayer water. Interlayer water is closely related to -OH groups. Materials rich in hydroxyl groups exhibit a strong adsorption capacity for heavy metals [27,28]. Therefore, the adsorption capacity of larger particle sizes is greater than that of smaller ones. Based on the aforementioned studies, it can be concluded that microwaves increase the interlayer water content of vermiculite, thereby enhancing the concentration of -OH groups and ultimately improving adsorption performance.

## 4. Conclusions

In summary, we investigated vermiculite with different particle sizes as the research subject and explored the changes in adsorption performance of Pb^2+^ after microwave irradiation. After microwave treatment, the larger particle size vermiculite exhibited higher adsorption performance. Although there were no significant differences in the specific surface area and pore size distribution among all samples, thermogravimetric quantitative analysis revealed that the interlayer water of the larger particle size was better retained. Fourier-transform infrared spectroscopy analysis also indicated that the -OH groups of the larger particle size demonstrated higher stability. The high content and stability of -OH groups may be the key factors contributing to the enhanced adsorption performance of Pb^2+^. This provides new insights for the preparation of hydroxyl-rich environmental adsorption materials.

## Figures and Tables

**Table 1:** Distribution of various components (wt. %) of vermiculite.

Component	SiO_2_	MgO	Al_2_O_3_	Fe_2_O_3_	K_2_O	Na_2_O	TiO_2_	CaO	F	BaO	LOI
Content	38.32	20.18	12.34	7.36	4.86	1.50	1.39	1.26	0.16	0.10	12.53

**Table 2:** Kinetics parameters for Pb^2+^ adsorption onto different sizes of expanded vermiculite.

	*q_e_*/ (mg·g^−1^)	Pseudo First Order Model			Pseudo Second Order Model		
		*q_e_*,_cal1_/ (mg·^−1^)	K_1_/min^−1^	R^2^	*q_e_*,_cal2_/ (mg·^−1^)	K_2_/(g·mg^−1^·min^−1^)	R^2^
Ver4	15.98	23.54	−1.84	0.84	15.42	−9.06 × 10^−3^	0.98
Ver2	12.87	18.49	−1.47	0.82	13.31	−1.35 × 10^−2^	0.96
Ver1	12.68	20.91	−1.39	0.63	13.19	−1.39 × 10^−2^	0.97
Ver0.5	11.01	18.99	−1.26	0.76	11.32	−1.74 × 10^−2^	0.97
Ver0.2	11.02	29.37	−1.49	0.64	11.36	−1.36 × 10^−2^	0.96

**Table 3:** The cation exchange capacity of the samples. Mmo/100 g.

Samples	K^+^	Na^+^	Ca^2+^	Mg^2+^	Ba^2+^	CEC (1)	CEC (2)
Ver	0.7734	31.64	1.427	0.1017	38.93	73.00	74.20
Ver4	0.8361	22.35	0.9300	0.0688	31.13	55.47	56.40
Ver2	0.9146	26.53	1.100	0.0791	31.79	60.57	64.60
Ver1	0.9893	27.18	1.114	0.0730	33.62	63.57	66.60
Ver0.5	0.8425	31.29	1.057	0.0807	35.56	68.98	67.40
Ver0.2	0.9087	27.27	0.9717	0.0865	41.55	70.94	73.20

CEC (1): Sum of six cation exchange volumes; CEC (2): Actual measured cation exchange capacity.

## Data Availability

The original contributions presented in the study are included in the article, further inquiries can be directed to the corresponding author.
